# Sphingosine kinase-1, S1P transporter spinster homolog 2 and S1P2 mRNA expressions are increased in liver with advanced fibrosis in human

**DOI:** 10.1038/srep32119

**Published:** 2016-08-26

**Authors:** Masaya Sato, Hitoshi Ikeda, Baasanjav Uranbileg, Makoto Kurano, Daisuke Saigusa, Junken Aoki, Harufumi Maki, Hiroki Kudo, Kiyoshi Hasegawa, Norihiro Kokudo, Yutaka Yatomi

**Affiliations:** 1 Department of Clinical Laboratory Medicine, The University of Tokyo, 7-3-1 Hongo, Bunkyo-ku, Tokyo Japan; 2 CREST, JST, Japan; 3 Department of Integrative Genomics, Tohoku Medical Megabank Organization, 2-1 Seiryo machi, Aobaku Sendai, Miyagi, Japan; 4 Graduate School of Pharmaceutical Science, Tohoku University, 6-3, Ara-makiazaaoba, Aobaku, Sendai, Miyagi, Japan; 5 Hepato-Biliary-Pancreatic Surgery Division, Department of Surgery, The University of Tokyo, Tokyo, Japan

## Abstract

The role of sphingosine 1-phosphate (S1P) in liver fibrosis or inflammation was not fully examined in human. Controversy exists which S1P receptors, S1P1 and S1P3 vs S1P2, would be importantly involved in its mechanism. To clarify these matters, 80 patients who received liver resection for hepatocellular carcinoma and 9 patients for metastatic liver tumor were enrolled. S1P metabolism was analyzed in background, non-tumorous liver tissue. mRNA levels of sphingosine kinase 1 (SK1) but not SK2 were increased in livers with fibrosis stages 3–4 compared to those with 0–2 and to normal liver. However, S1P was not increased in advanced fibrotic liver, where mRNA levels of S1P transporter spinster homolog 2 (SPNS2) but not S1P-degrading enzymes were enhanced. Furthermore, mRNA levels of S1P2 but not S1P1 or S1P3 were increased in advanced fibrotic liver. These increased mRNA levels of SK1, SPNS2 and S1P2 in fibrotic liver were correlated with α-smooth muscle actin mRNA levels in liver, and with serum ALT levels. In conclusion, S1P may be actively generated, transported to outside the cells, and bind to its specific receptor in human liver to play a role in fibrosis or inflammation. Altered S1P metabolism in fibrotic liver may be their therapeutic target.

Liver fibrosis results from chronic liver injury, and is characterized by excessive deposition of collagen and other components of extracellular matrix. Although the exact mechanism of liver fibrosis still remains to be clarified, sphingosine 1-phosphate (S1P) is now assumed to be among pivotal regulators of liver fibrosis^1^. Many biological actions of S1P as an extracellular mediator are generated through binding to G-protein-coupled S1P receptors 1–5 2. S1P1-3 are widely expressed in a variety of tissues including liver, whereas S1P4 is exclusively found on lymphoid and hematopoietic tissue and S1P5 is mainly expressed in the central nervous system^3^. To bind S1P receptors, a large amount of S1P may be secreted to the extracellular space, where the export of S1P from the cells requires the action of S1P transporter spinster homolog 2 (SPNS2)^4^,^5^,^6^,^7^. To generate S1P inside the cells, sphingosine kinases (SKs) are known to be a key enzyme with two isoforms (SK1 and SK2), differing in sequence, catalytic properties, localization, and functions^8^.

Hepatic stellate cells (HSCs) are known to play a central role in the development of liver fibrosis, undergoing phenotypic change to myofibroblast-like cells in the process of liver fibrosis^9^,^10^,^11^. We first found that S1P stimulated rat HSC proliferation via an extracellular mechanism *in vitro*^12^, and later we further observed that liver fibrosis caused by repeated administration of carbon tetrachloride was reduced in S1P receptor 2-deficient mice *in vivo*^13^. Moreover, S1P has a stimulatory role in portal hypertension^14^, where S1P2 antagonist is effective in reducing portal vein pressure in rodents^15^. In contrast, an important role of S1P1 and S1P3 but not S1P2 in liver fibrosis has been demonstrated in mice^16^. Furthermore, the contribution of intracellular S1P to liver fibrosis, independent of its receptors, has been recently reported in human^17^. Although these lines of evidence suggest that S1P plays an important role in pathophysiology of liver fibrosis, the contribution of its receptors to liver fibrosis remains elusive. Furthermore, the mode of action of S1P in liver fibrosis may vary with species examined. If so, a role of S1P in liver fibrosis should be examined in human in order to establish therapeutic strategy for liver fibrosis by regulating the effect of S1P. In the current study, we sought to examine the expressions of S1P and its metabolites and receptors, and the enzymes in its metabolic pathway in human liver tissue with various stages of fibrosis.

## Results

### Patient characteristics

A total of 80 hepatocellular carcinoma (HCC) patients who underwent a surgical treatment were enrolled. Clinical and laboratory characteristics of the patients are shown in Table 1. There were 61 male and 19 female patients with a median age of 71 years. Fourteen patients (17.5%) were with hepatitis B virus infection, and 32 patients (40.0%) with hepatitis C virus infection. Seventy-eight (97.5%) of the patients was with Child-Pugh class A, and fibrosis stage 4 in the background liver was observed in 26 patients (32.5%).

### Alterations of sphingolipid metabolites and the related enzymes in human fibrotic liver

Table 2 shows the associations between histological fibrosis stage and mRNA expressions or levels of sphingolipid metabolites and the related enzymes in the background, non-tumorous liver tissues. SK1 mRNA expression levels in liver were significantly higher in patients with advanced liver fibrosis (*P* = 0.005, after adjustment for age and sex); the median mRNA expression level of SK1 in livers with fibrosis stages 0–2 and 3–4 were 1.79 × 1/10^5^ (1.21 × 1/10^5^–3.41 × 1/10^5^) and 3.67 × 1/10^5^ (2.02 × 1/10^5^–6.63 × 1/10^5^), respectively. In contrast, SK2 mRNA expression levels were not different between livers with fibrosis stages 0–2 and those with 3–4. In spite of the increased mRNA levels of SK1, catalyzing the synthesis of S1P from sphingosine, S1P levels as well as Sph levels were not different between livers with fibrosis stages 0–2 and those with 3–4. Then, regarding a degrading enzyme of S1P, both S1P lyase (SPL) and S1P phosphatase 1 (SPP1) mRNA expression levels were not different between livers with fibrosis stages 0–2 and those with 3–4, while mRNA expression levels of SPNS2, a transporter of S1P from inside the cells to outside, were significantly higher in livers with fibrosis stages 3–4 than in those with 0–2 (*P* = 0.03, after adjustment for age and sex); the median mRNA expression level of SPNS2 in livers with fibrosis stages 0–2 and 3–4 were 1.84 × 1/10^5^ (1.43 × 1/10^5^–2.54 × 1/10^5^) and 2.38 × 1/10^5^ (1.89 × 1/10^5^–3.68 × 1/10^5^), respectively. Among S1P receptors, S1P2 mRNA expression levels were higher in livers with fibrosis stages 3–4 than in those with 0–2 (*P* = 0.04, univariate analysis), while S1P1 and S1P3 mRNA levels were not different between livers with fibrosis stages 0–2 and those with 3–4. Collectively, our analysis of mRNA levels showed increased expression of SK1, SPNS2 and S1P2 in livers with fibrotic stages 3–4 compared to those with 0–2, and the similar trend to mRNA levels was observed in protein levels of SK1, SPNS2 and S1P2 analyzed by Western blot using the residual liver tissues (Supplementary Figure 1). These results suggest that inside-out signaling^18^ of S1P may operate actively in fibrotic liver, and in line with this a strong correlation between SPNS2 and S1P2 mRNA levels in liver was observed (Spearman’s rho = 0.6057, *P* < 0.001, data not shown). We also investigated the associations between histological fibrosis stages and mRNA expressions or levels of sphingolipid metabolites and the related enzymes in patients with hepatitis B, with hepatitis C, or without hepatitis viruses (Supplementary Tables 1–3). Although statistically significant differences were not observed, SK1, SPNS2, and S1P2 mRNA levels were higher in patients with fibrosis stages 3–4 compared to 0–2 in all etiologies (Supplementary Figure 2).

**Figure 1:**
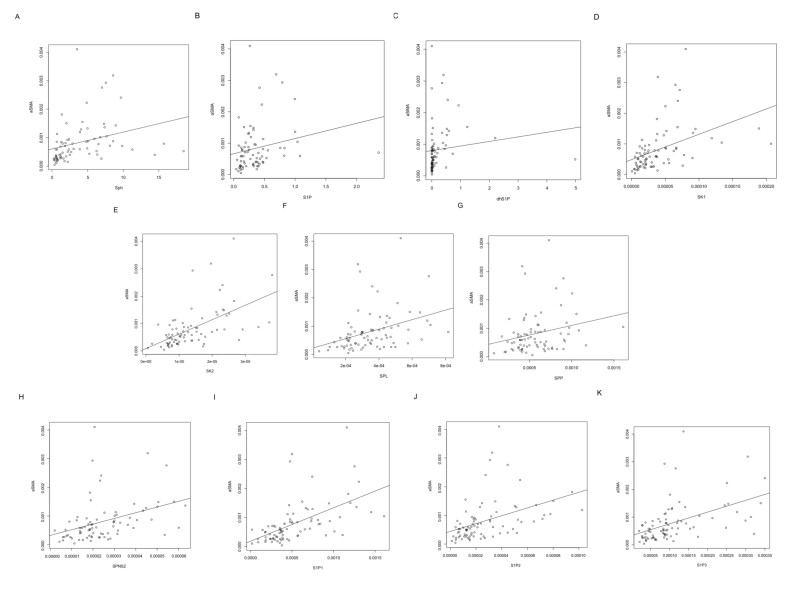
Relationship between α-SMA mRNA expression and mRNA expressions (or levels) of Sph (**A**), S1P (**B**), dhS1P (**C**), SK1 (**D**) SK2 (**E**), SPL (**F**), SPP (**G**), SPNS2 (**H**), S1P1 (**I**), S1P2 (**J**), and S1P3 (**K**) in liver tissue. A significant correlations were observed between α-SMA mRNA expression and mRNA expression/levels of Sph (spearman’s rank correlation coefficient; ρ = 0.57, *P* < 0.001), S1P (spearman’s rank correlation coefficient; ρ = 0.34, *P* = 0.003), dhS1P (spearman’s rank correlation coefficient; ρ = 0.41, *P* < 0.001), SK1 (spearman’s rank correlation coefficient; ρ = 0.60, *P* < 0.001), SK2 (spearman’s rank correlation coefficient; ρ = 0.62, *P* < 0.001), SPL (spearman’s rank correlation coefficient; ρ = 0.46, *P* < 0.001), SPP1 (spearman’s rank correlation coefficient; ρ = 0.31, *P* = 0.006), SPNS2 (spearman’s rank correlation coefficient; ρ = 0.51, *P* < 0.001), S1P1 (spearman’s rank correlation coefficient; ρ = 0.68, *P* < 0.001), S1P2 (spearman’s rank correlation coefficient; ρ = 0.58, *P* < 0.001), or S1P3 (spearman’s rank correlation coefficient; ρ = 0.60, *P* < 0.001).

Then, to investigate whether the alterations of sphingolipid metabolites and the related enzymes in fibrotic liver might be associated with HSCs as a central player in liver fibrosis, we measured mRNA levels of α-smooth muscle actin (α-SMA), a marker of HSCs with phenotypic change to myofibroblast-like cells. As depicted in Fig. 1, mRNA levels of α-SMA were significantly correlated with Sph, S1P and dhS1P levels, and mRNA levels of SK1, SK2, SPL, SPP1 or SPNS2 in liver. Furthermore, mRNA levels of S1P1, S1P2, and S1P3 were significantly correlated with α-SMA mRNA levels in liver (Fig. 1F–H). Collectively, there may be a link between the altered S1P metabolism in human fibrotic liver and HSCs.

Finally, in order to compare normal liver with advanced fibrotic liver in human from the viewpoint of sphingolipid metabolites and the related enzymes, we have obtained normal liver samples from the patients undergoing hepatic resection for metastasis from colorectal cancer, although the number of patients was small (n = 9). The clinical and laboratory characteristics of these patients are shown in the Supplementary Table 4, and mRNA expression or levels of sphingolipid metabolites and the related enzymes in normal liver and advanced fibrotic liver are shown in the Supplementary Table 5. In line with the comparison between liver fibrosis stages 0–2 and 3–4, the higher SK1, SPNS2, and S1P2 mRNA levels were noted in the patients with fibrosis stages 3–4 compared to the patients with normal liver, though not statistically significant due to the relatively small number of patients with normal liver.

### Relationships of sphingolipid metabolites and the related enzymes in human fibrotic liver with clinical parameters

Then, the relationships between sphingolipid metabolites and the related enzymes in human fibrotic liver and clinical parameters were examined. Table 3 shows the relationship of sphingosine metabolites and the related enzymes with clinical parameters. There was no association between S1P levels in liver and clinical parameters. On the other hand, among S1P-related enzymes in liver, SK1 mRNA levels were correlated with serum aspartate aminotransferase (AST), alanine aminotransferase (ALT) and total bilirubin levels, SK2 with serum γ-glutamyl transpeptidase (GGT) levels, SPL, SPP1 and SPNS2 with serum ALT levels. Furthermore, S1P1, S1P2 and S1P3 mRNA levels in liver were correlated with serum ALT levels.

Regarding the etiology of liver diseases, SPL and SPNS2 mRNA levels in liver were lower in the patients with hepatitis B virus infection than in those with other etiology (data not shown).

## Discussion

Although S1P has been shown to play an important role in liver fibrosis or inflammation in rodents^1^,^19^,^20^, the clinical roles of S1P and its metabolites in liver fibrosis or inflammation have not been fully elucidated in human. Here, we examined the relationship between the severity of liver fibrosis/inflammation and mRNA expressions and/or levels of sphingolipid metabolites and the related enzymes using surgically-resected human liver tissues. In the current study, mRNA expression of SK1 but not SK2 was enhanced in fibrotic liver in human, in line with the previous report^21^, however, S1P, generated with catalytic action of SK1, was not increased in human fibrotic liver. There were at least two possibilities to explain these findings, increased degradation and/or export of S1P. As a result, mRNA expression of SPL or SPP1, an enzyme to degrade S1P, was not altered, while mRNA expression of SPNS2, a transporter of S1P, was enhanced in human fibrotic liver. Furthermore, mRNA expression of S1P2 among S1P receptors was enhanced in fibrotic liver in human. These results suggest that S1P, generated actively by SK1 in human fibrotic liver, may be transported to the outside of liver cells by SPNS2, and that the exported S1P may bind to S1P2 to exert its action by an extracellular mechanism.

Controversy exits regarding the contribution of S1P receptors to liver fibrosis. The enhanced mRNA expression of S1P2 has been previously reported in fibrotic liver in rodents^15^, while S1P2 expression were not altered in liver fibrosis in rodents^16^ but massively decreased in human^21^. The current finding in human is in line with the former evidence in rodents, which showed up-regulation of S1P2 in fibrotic liver, and may suggest a rationale to establish therapeutic strategy for liver fibrosis by modifying S1P action based on the evidence in rodents. In this regard, we^13^ and others^19^,^22^ have reported an important role of S1P/S1P2 axis in liver fibrosis using *S1P2*-deficient mice or S1P2 antagonist in rodents. Thus, antagonism of S1P2 merits consideration as therapeutic strategy for liver fibrosis in human. On the other hand, the overexpression of S1P1 and S1P3 has been reported in human fibrotic liver tissue^21^. The reasons of these discrepancies have not been solved yet, and further studies with more samples may be useful to find a precise role of S1P receptors in liver fibrosis.

To the best of our knowledge, this is the first report showing the altered expression of SPNS2 in liver. It should be further elucidated whether enhanced expression of SPNS2 in liver might be simply associated with liver fibrosis or importantly involved in its mechanism. Although a gain- and loss-of-function approach may be useful to clarify how SPNS2 sustains fibrogenic process in liver, no phenotype has been reported in liver in *SPNS2*-knockout mice so far^7^. Thus, a role of SPNS2 in liver fibrosis should be further elucidated. Of interest is a fact that a mutation in SPNS2 caused similar phenotype to that in S1P2, a defect in heart development in zebrafish^4^. Furthermore, SPNS2 deficiency^23^ and S1P2 deficiency^24^ caused similarly hearing loss in mice. These lines of evidence may suggest a crucial link between SPNS2 and S1P2, when S1P exerts its effect. A strong correlation between mRNA levels of SPNS2 and S1P2 in fibrotic liver observed in the current study may be in line with this speculation.

We have recently shown in HCC, which frequently develops in fibrotic liver, the increased mRNA levels in SK1, SK2 and SPL but not altered mRNA levels in SPNS2 and the rather reduced levels of S1P compared to non-HCC tissues^25^. These findings and the current ones may suggest active inside-out signaling of S1P in fibrotic liver, while enhanced degradation of S1P in HCC, among which crucial events involved in fibrogenesis and carcinogenesis in liver should be further elucidated.

We previously reported that plasma S1P levels were reduced in patients with chronic hepatitis C in association with liver fibrosis^26^, which may not be consistent with the current results. Although the local concentration of S1P in the outside of liver cells of fibrotic liver may be increased with enhanced expressions of SK1 and SPNS2, the exported S1P by inside-out signaling^18^ may be bound to S1P receptors and taken up by liver cells, leading to reduced S1P levels in blood stream.

The SK1/S1P pathway has been reportedly implicated also in inflammation mediated by TNF-α^27^. Other inflammatory signaling molecules such as IFN-λ, IL-1β, IgE^28^ have been also shown to activate SK1, further suggesting the importance of the SK1/S1P pathway in the inflammatory response. In another study, *SK1*-knockout mice had decreased tissue SK activity and a 50% decrease in serum S1P levels, but not significant difference in tissue S1P under normal conditions^29^. In the present study, in spite of positive correlation between serum ALT levels and SK1/SPNS2/S1PRs expressions, serum ALT levels were not correlated with S1P/dhS1P levels in liver tissue. Taken together the above studies and the present study, inflammation and recruitment of inflammatory cells are important in liver disease progression and are, at least in part, mediated by the SK1 and extracellular secretion of S1P via SPNS2. Although higher SK1 expression results in increased S1P concentration in liver cells, intracellular S1P may be reduced by up-regulated SPNS2; and a larger amount of S1P may be secreted to extracellular space to bind S1PRs in patients with active inflammation.

Among the enzymes in the S1P metabolic pathway, acidic sphingomyelinase to generate ceramide was reportedly associated with HSC activation, and its mRNA levels were enhanced in liver samples from patients with nonalcoholic steatohepatitis compared in those from healthy subjects^30^. On the other hand, acidic sphingomyelinase mRNA levels were not significantly enhanced in fibrotic liver in the current study (data not shown). Collectively, it is speculated that acidic sphingomyelinase mRNA expression in liver might be stimulated by steatosis rather than fibrosis. Nonetheless, a role of acidic sphingomyelinase in liver fibrosis should be further elucidated.

Some limitations of this study should be acknowledged. Firstly, because we used background liver samples of HCC patients or patients with liver metastasis, the current results may be affected by the presence of HCC^31^ or other liver tumors. However, it is difficult due to the ethical concerns to obtain liver specimen from patients without HCC or other liver tumors, because the vast majority of patients who undergo liver resection are HCC patients or patients with liver metastasis in our hospital. As the second concern, our results may be affected by hepatic decompensation^32^. However, its effect may be minimized in the present study, because almost all (97.5%) patients were assumed to have well preserved liver function (Child-Pugh classification A). Thirdly, SPL and SPNS2 mRNA levels in liver were lower in the patients with HBV infection than in those with other etiology in the present study, suggesting that it would be interesting to analyze the samples separately according to the etiology of liver diseases. The other studies reported difference of serum sphingolipid profiles among patients with hepatitis C, hepatitis B, and non alcoholic fatty liver disease^33^,^34^. In the results of separate analyses according to etiologies (Supplementary Tables 1–3), almost the same tendencies as the results in whole patients were observed in all etiologies (Supplementary Figure 2). However, the sample size in each etiology was relatively small, being statistically underpowered. Further studies with larger samples using liver tissue without HCC such as liver biopsy samples may be worth trying.

In conclusion, our study, using human liver tissue samples, shows that increased activity of SK1/S1P pathway, S1P secretion via SPNS2, and S1P binding to S1P2 may play an important role in the progression of liver fibrosis with recruitment of inflammatory cells. Altered S1P metabolism in fibrotic liver may be a possible drug target for patients with chronic hepatitis or liver cirrhosis.

## Patients and Methods

### Subjects

The liver tissue samples analyzed in the present study were derived from the patients who were treated in the Hepatobiliary Pancreatic Surgery Division, Department of Surgery, the University of Tokyo Hospital between January 2013 and October 2014. Among them, sufficient liver tissue samples could be obtained in 80 patients to analyze the levels of sphingolipid metabolites and the related enzymes. All the enrolled patients underwent liver resection for the treatment of HCC. Additionally, we enrolled the patients undergoing hepatic resection for metastasis from colorectal cancer in above period to obtain normal liver samples (n = 9).

This study was carried out in accordance with the ethical guidelines of the 1975 Declaration of Helsinki and was approved by the Institutional Research Ethics Committee of the University of Tokyo. A written informed consent was obtained for the use of the samples.

### Quantitative real time PCR for enzymes in sphingolipid metabolism

Total RNA of tissue samples was extracted using TRIZOL reagent (Invitrogen, CA, USA). One microgram of purified total RNA was transcribed using a SuperScript^TM^ First-Strand Synthesis System for RT-PCR (Invitrogen). Quantitative real time PCR was performed with a LightCycler FastStart DNA Master SYBR Green I kit (Roche Molecular Diagnostics, CA, USA) or TaqMan Universal Master Mix. The primer pairs used were as follows: human SK1: 5′-CTGGCAGCTTCCTTGAACCAT-3′ and 5′-TGTGCAGAGACAGCAGGTTCA-3′; human SK2: 5′-CCAGTGTTGGAGAGCTGAAGGT-3′ and 5′-GTCCATTCATCTGCTGGTCCTC-3′; human SPL: 5′-GCCAGAGAGTTTATGGTCAAGGTT-3′ and 5′-CAACTTGTCTTGAATCTTACGACCAA-3′; human SPP1: 5′-GCCGCTGGCAGTACCCT-3′ and 5′-AATAGAGTGCATTCCCATGTAAATTCT-3′; internal control ribosomal 18s: 5′-GTAACCCGTTGAACCCCATT-3′ and 5′-CCATCCAATCGGTAGTAGCG-3′; human S1P receptor 1 (S1P1): 5′-CCTCTTCCTGCTAATCAGCG-3′ and 5′-ACAGGTCTTCACCTTGCAGC-3′; human S1P receptor 2 (S1P2): 5′-GCCTAGCCAGTTCTGAAAGC-3′ and 5′-ACAGGTACATTGCCGAGTGG-3′; human S1P receptor 3 (S1P3): 5′-CGACGGAGGAGCCCTTTTTC-3′ and 5′-TCCAAAATCCACGAGAGGGC-3′; acid sphingomyelinase: 5′-AAGCCCTGCGCACCCTCAGAA-3′ and 5′-CCTGAAGCTCCCCCACCAGCC-3′; α-SMA: 5′-CAGCCAAGCACTGTCAGGAAT-3′ and 5′-TTTGCTCTGTGCTTCGTCAC-3′. The samples were incubated for 10 min at 95 °C, followed by 40 cycles at 95 °C for 15 sec, 60 °C for 1 min. The target gene mRNA expression level was relatively quantified to ribosomal 18 s using 2^−ΔΔCt^ method (Applied Biosystems, User Bulletin No. 2).

### Measurement of sphingolipids in liver tissues

Sample preparation procedure was referenced by previously reported^35^. Liver (approximately 10–50 mg) was placed into a sample tube (2.0 mL) and snap frozen in liquid nitrogen. Two-hundred microliters of formic acid (0.1% (volume fraction) in methanol) containing the internal standard solution (10.0 ng/mL, mixture of sphingosine (Sph) 17:1 and S1P 17:1) was added to the frozen sample. The mixtures were homogenized using a lysis and homogenization system (Precellys) (5,000 × rpm, 15 s, 1 Zr bead). The samples were then centrifuged at 16,400 × g for 20 min at 4 °C, and the supernatants were passed through a 96-well plate for deproteinization (Sirocco, Waters). Aliquots (5 μL) of the deproteinized samples were analyzed by liquid chromatography-tandem mass spectrometry (LC-MS/MS). The LC-MS/MS system consists of a NANOSPACE SI-II HPLC (Shiseido) and a TSQ Quantum Ultra triple quadrupole MS (Thermo Fisher Scientific) equipped with a heated-electrospray ionization-II source. Sphingolipids were analyzed by LC-MS/MS in the selected reaction monitoring mode, and the optimal condition was described elsewhere^25^. The ratio of the peak area of the analyte to the internal standard was used for quantification with the calibration curves (0.05–100 ng/mL) by each standard solutions.

### Immunoblot analysis

Tissue extracts were prepared by using M-PER^®^ Mammalian Protein Extraction Reagent (Thermo Fisher Scientific Inc., IL, USA) plus Halt^TM^ Protease Inhibitor Cocktail (Thermo Fisher Scientific Inc.). Immunoblot analysis was performed using NuPAGE SDS-PAGE Gel (Invitrogen) and iBlot Dry Blotting System (Invitrogen) with specific antibodies against SK1 (Cell Signaling Technology, MA, USA) dilution 1:1000, SPNS2 (Abcam, Cambridge, UK) dilution 1:150, S1P2 (Novusbio, Minneapolis, USA) dilution 1ug/ml and beta-actin (Sigma-Aldrich, St. Louis Missouri, USA) dilution 1:2000. Immunoreactive proteins were visualized using a chemiluminescence kit (GE Healthcare, Buckinghamshire, UK), and recorded using a LAS-4000 image analyzer (Fuji Film, Tokyo, Japan).

### Study end-points

We analyzed the relationship between histological fibrosis stages and mRNA expressions/levels of sphingolipid metabolites and the related enzymes in liver tissues. We also examined the relationship between sphingolipid metabolites and the related enzymes and serological or other biological markers. The histological stages of fibrosis were assessed using the reproducible METAVIR scoring system^36^,^37^.

### Statistical analysis

Continuous variables were presented as median with 1^st^ and 3^rd^ percentiles while categorical variables were expressed as frequencies (%). For comparison between two groups, Mann-Whitney U test was used; and for comparison of three groups, Kruskal-Wallis test was used. Multiple linear regression model analyses were used to adjust the contribution of fibrosis by other covariates such as sex or age. The potential associations between mRNA expressions and following factors were assessed using Spearman’s rank correlation coefficient: age, platelet count, serum albumin, aspartate aminotransferase (AST), alanine aminotransferase (ALT), γ-glutamyl transpeptidase (GGT), alkaline phosphatase (ALP), total bilirubin, prothrombin time (PT), and mRNA level of α-smooth muscle actin (α-SMA). All statistical analyses were two-sided, and the threshold of the reported *P* values for significance was accepted as <0.05. All statistical analyses were performed using R statistic software version 3.1.0 (http://www.r-project.org).

## Additional Information

**How to cite this article**: Sato, M. *et al.* Sphingosine kinase-1, S1P transporter spinster homolog 2 and S1P2 mRNA expressions are increased in liver with advanced fibrosis in human. *Sci. Rep.* **6**, 32119; doi: 10.1038/srep32119 (2016).

## Supplementary Material

Supplementary Information

## Figures and Tables

**Table 1:** Patient characteristics.

Parameter	n = 80
Female/Male	19/61
Age (years)	71 (65–75)
Types of hepatitis	
Hepatitis B (%)	14 (17.5)
Hepatitis C (%)	32 (40.0)
Nonb nonC (%)	34 (42.5)
Fibrosis stage 0/1/2/3/4	7/12/14/21/26
Child-Pugh classification	
A (%)	78 (97.5)
B (%)	2 (2.5)
C (%)	0 (0)
Platelet count (×10^4^ /μL)	15.5 (12.3–19.3)
Albumin (g/dL)	4.0 (3.7–4.2)
AST (U/L)	32 (25–50)
ALT (U/L)	28 (20–48)
GGT (U/L)	48 (29–87)
ALP (U/L)	251 (204–322)
Total bilirubin (mg/dL)	0.7 (0.6–0.9)
PT (%)	100 (96–100)

Continuous variables were represented as the median with 1^st^ and 3^rd^ percentiles and categorical variables were as number and frequencies (%).

AST: aspartate aminotransferase, ALT: alanine aminotransferase, GGT: γ-glutamyl transpeptidase, ALP: alkaline phospatase, PT: prothrombin time.

**Table 2:** Associations between fibrosis stage and mRNA expression or levels of sphingolipid metabolites and the related enzymes in liver tissues (n = 80).

Variable	Median/Number (1^st^–3^rd^ Quartile)		*P* values	
	F0–2	F3–4	*P* value	Adjusted *P* value*
Sph (ng/mg)	2.18 (1.16–5.02)	2.87 (1.08–6.90)	0.79	0.51
S1P (ng/mg)	0.290 (0.137–0.391)	0.299 (0.174–0.478)	0.31	0.11
dhS1P (ng/mg)	0.0190 (0.00675–0.0678)	0.0415 (0.00600–0.327)	0.49	0.92
SK1 (×1/10^5^)	1.79 (1.21–3.41)	3.67 (2.02–6.63)	0.003	0.005
SK2 (×1/10^5^)	0.938 (0.783–1.67)	1.18 (0.871–1.88)	0.40	0.42
SPL (×1/10^4^)	3.12 (2.45–4.31)	3.52 (2.23–4.48)	0.91	0.72
SPP1 (×1/10^4^)	5.97 (4.12–7.69)	5.00 (3.98–6.77)	0.36	0.56
SPNS2 (×1/10^5^)	1.84 (1.43–2.54)	2.38 (1.89–3.68)	0.01	0.03
S1P1 (×1/10^4^)	3.85 (2.77–6.22)	4.47 (3.02–7.33)	0.45	0.45
S1P2 (×1/10^5^)	1.53 (0.896–3.12)	2.42 (1.53–3.84)	0.04	0.12
S1P3 (×1/10^5^)	8.35 (4.51–10.1)	9.37 (5.59–15.8)	0.08	0.08

^*^Adjusted for sex and patients age (independent variables). The dependent variables of each *P* value are the items in the leftmost fields of the corresponding row (Sph, S1P, SK1, *etc*.).

Sph, sphingosine; S1P, sphingosine-1-phosphate; SK, sphingosine kinase; SPL, sphingosine-1-phosphate lyase; SPP1, sphingosine-1-phosphate phosphatase 1; SPNS2, S1P transporter spinster homolog 2; S1P1, sphingosine 1-phosphate receptor 1; S1P2, sphingosine 1-phosphate receptor 2; S1P3, sphingosine 1-phosphate receptor 3.

**Table 3:** Relationships of sphingolipid metabolites and the related enzymes with each clinical parameter.

Parameter	Sph		S1P		dhS1P		
	Spearman’s rho	*P* value	Spearman’s rho	*P* value	Spearman’s rho	*P* value	
*Sex		0.15		0.76		0.85	
Age (years)	0.0650	0.58	−0.0285	0.81	−0.0556	0.64	
Platelet count (×10^4^/μL)	0.0406	0.73	−0.0481	0.68	0.0327	0.78	
Albumin (g/dL)	0.1206	0.31	0.0934	0.43	0.0173	0.88	
AST (U/L)	0.0780	0.51	0.0353	0.77	0.2387	0.04	
ALT (U/L)	0.1678	0.15	0.0434	0.71	0.2504	0.03	
GGT (U/L)	0.0010	0.93	−0.1471	0.21	0.1497	0.20	
ALP (U/L)	−0.0741	0.53	−0.0157	0.89	0.1717	0.14	
Total bilirubin (mg/dL)	0.2019	0.08	0.1244	0.29	0.2182	0.06	
PT%	0.0803	0.50	−0.2055	0.08	−0.1854	0.11	
**SK1**		**SK2**		**SPL**		**SPP1**	
**Spearman’s rho**	***P*** **value**	**Spearman’s rho**	***P*** **value**	**Spearman’s rho**	***P*** **value**	**Spearman’s rho**	***P*** **value**
	0.17		0.85		0.21		0.25
−0.1114	0.33	0.1703	0.13	−0.0132	0.91	−0.2161	0.054
−0.1709	0.13	−0.0320	0.78	−0.1241	0.27	0.0077	0.95
0.1565	0.17	0.0864	0.45	0.0836	0.46	0.2433	0.03
0.3107	0.005	0.1048	0.35	0.1477	0.19	0.0796	0.48
0.4031	<0.001	0.1524	0.18	0.2491	0.03	0.2268	0.04
−0.0241	0.83	−0.2675	0.02	−0.2143	0.06	−0.0844	0.46
0.0697	0.54	−0.0237	0.98	−0.0211	0.85	−0.0114	0.92
0.2118	0.048	0.0170	0.88	−0.009	0.94	−0.0058	0.96
−0.1975	0.08	0.0062	0.96	−0.1176	0.30	−0.0349	0.76
**SPNS2**		**S1P1**		**S1P2**		**S1P3**	
**Spearman’s rho**	***P*** **value**	**Spearman’s rho**	***P*** **value**	**Spearman’s rho**	***P*** **value**	**Spearman’s rho**	***P*** **value**
	0.42		0.76		0.15		0.24
0.0756	0.50	0.0254	0.82	0.0287	0.80	−0.0619	0.59
−0.1368	0.23	−0.0470	0.68	−0.1361	0.23	−0.1034	0.36
0.0699	0.54	0.1704	0.13	0.1149	0.31	0.2635	0.02
0.1529	0.18	0.1751	0.12	0.1848	0.10	0.2007	0.07
0.3024	0.006	0.2875	0.01	0.2699	0.01	0.2845	0.01
−0.1840	0.10	−0.1392	0.22	−0.1818	0.11	−0.0137	0.90
−0.0585	0.61	−0.0352	0.76	−0.0910	0.42	0.0237	0.83
−0.1347	0.91	0.0633	0.58	0.0454	0.69	−0.0058	0.96
−0.0503	0.66	0.0016	0.99	−0.0666	0.56	−0.6647	0.56

^*^Relation of each variable with sex is analyzed using Mann-Whitney U test.

Sph, sphingosine; S1P, sphingosine-1-phosphate; SK, sphingosine kinase; SPL, sphingosine-1-phosphate lyase; SPP1, sphingosine-1-phosphate phosphatase 1; SPNS2, S1P transporter spinster homolog 2; S1P1, sphingosine 1-phosphate receptor 1; S1P2, sphingosine 1-phosphate receptor 2; S1P3, sphingosine 1-phosphate receptor 3; AST, aspartate aminotransferase; ALT, alanine aminotransferase; GGT, γ-glutamyl transpeptidase; ALP, alkaline phosphatase; PT, prothrombin time.
