# Supplementary material for: Sphingosine kinase-1, S1P transporter spinster homolog 2 and S1P2 mRNA expressions are increased in liver with advanced fibrosis in human
Source: Sci Rep. 2016 Aug 26;6:32119. doi: 10.1038/srep32119 (PMC4999825; doi:10.1038/srep32119)
Supplement: Supplementary Information [file srep32119-s1.pdf]

Sphingosine kinase-1, S1P transporter spinster homolog 2 and S1P2 mRNA expressions are increased in liver with advanced fibrosis in human.

Masaya Sato,[1] Hitoshi Ikeda,[1, 5] Baasanjav Uranbileg,[1] Makoto Kurano,[1, 5] Daisuke Saigusa,[3, 5] Junken Aoki,[4, 5] Harufumi Maki,[2] Hiroki Kudo,[2] Kiyoshi Hasegawa,[2] Norihiro Kokudo,[2] Yutaka Yatomi[1, 5]

[1]Department of Clinical Laboratory Medicine and [2]Hepato-Biliary-Pancreatic Surgery Division, Department of Surgery, The University of Tokyo, Tokyo, [3]Department of Integrative Genomics, Tohoku Medical Megabank Organization, and [4]Graduate School of Pharmaceutical Sciences, Tohoku University, Miyagi, and [5]CREST, JST, Japan

Running Head

S1P metabolism in liver fibrosis

Corresponding Author:

Hitoshi Ikeda, M.D., Ph.D.

Department of Clinical Laboratory Medicine,

Graduate School of Medicine, The University of Tokyo

7-3-1 Hongo, Bunkyo-ku, Tokyo 113-8655, Japan

TEL +81-3-3815-5411

FAX +81-3-3814-0021

e-mail: ikeda-1im@h.u-tokyo.ac.jp

Supplementary Dataset

Supplementary Table 1. Associations between fibrosis stage and mRNA expression or levels of sphingolipid metabolites and the related enzymes in liver tissues in patients with HBV (n=14).

Variable	Median/Number (1st-3rd Quartile)		*P* values	
	F0-2	F3-4	*P* value	Adjusted *P* value*
Sph (ng/mg)	1.54 (0.880-4.15)	1.19 (0.507-4.94)	0.59	0.97
S1P (ng/mg)	0.345 (0.166-0.381)	0.193 (0.178-0.303)	0.70	0.30
dhS1P (ng/mg)	0.0170 (0.00450-0.0355)	0.0190 (0.00525-0.370)	0.87	0.92
SK1 ($\times 1/10^5$)	1.95 (1.41-3.03)	2.39 (0.804-3.70)	0.95	0.97
SK2 ($\times 1/10^5$)	0.847 (0.675-1.03)	0.878 (0.643-1.27)	0.75	0.85
SPL ($\times 1/10^4$)	2.33 (2.13-3.50)	2.23 (2.19-2.45)	0.57	0.26
SPP1 ($\times 1/10^4$)	6.62 (4.45-7.59)	3.91 (3.15-4.85)	0.14	0.06
SPNS2 ($\times 1/10^5$)	0.979	1.65	0.66	0.88

	(0.854-2.00)	(1.12-2.11)		
S1P1 (×1/10^4)	3.45	2.86	0.34	0.30
	(2.88-3.71)	(1.78-3.88)		
S1P2 (×1/10^5)	1.17	1.38	0.66	0.74
	(0.832-2.24)	(1.07-2.62)		
S1P3 (×1/10^5)	4.95	6.69	0.75	0.49
	(3.99-7.62)	(4.28-8.72)		

*Adjusted for sex and patients age (independent variables). The dependent variables of each *P* value are the items in the leftmost fields of the corresponding row (Sph, S1P, SK1, *etc*.).

HBV, hepatitis B virus; Sph, sphingosine; S1P, sphingosine-1-phosphate; SK, sphingosine kinase; SPL, sphingosine-1-phosphate lyase; SPP1, sphingosine-1-phosphate phosphatase 1; SPNS2, S1P transporter spinster homolog 2; S1P1, sphingosine 1-phosphate receptor 1; S1P2, sphingosine 1-phosphate receptor 2; S1P3, sphingosine 1-phosphate receptor 3

Supplementary Table 2. Associations between fibrosis stage and mRNA expression or levels of sphingolipid metabolites and the related enzymes in liver tissues in patients with HCV (n=32).

Variable	Median/Number (1st-3rd Quartile)		*P* values	
	F0-2	F3-4	*P* value	Adjusted *P* value*
Sph (ng/mg)	2.87 (1.14-6.37)	2.70 (1.29-6.52)	0.96	0.68
S1P (ng/mg)	0.315 (0.155-0.373)	0.319 (0.167-0.759)	0.72	0.51
dhS1P (ng/mg)	0.0240 (0.0140-0.155)	0.0490 (0.0130-0.230)	0.98	0.85
SK1 ($\times 1/10^5$)	2.06 (1.01-3.90)	5.58 (2.59-7.81)	0.02	0.06
SK2 ($\times 1/10^5$)	0.983 (0.804-1.59)	1.41 (0.968-2.24)	0.43	0.30
SPL ($\times 1/10^4$)	3.05 (2.94-3.18)	4.01 (2.74-4.87)	0.34	0.20
SPP1 ($\times 1/10^4$)	4.16 (4.12-4.43)	5.34 (4.38-7.09)	0.17	0.22
SPNS2 ($\times 1/10^5$)	1.82 (1.52-2.71)	3.11 (1.89-4.39)	0.15	0.25

S1P1 ($\times 1/10^4$)	3.85 (2.92-4.98)	4.96 (3.82-8.83)	0.16	0.24
S1P2 ($\times 1/10^5$)	1.39 (0.896-3.12)	3.32 (1.60-5.13)	0.08	0.13
S1P3 ($\times 1/10^5$)	8.49 (5.27-9.72)	10.7 (7.28-14.2)	0.23	0.74

*Adjusted for sex and patients age (independent variables). The dependent variables of each *P* value are the items in the leftmost fields of the corresponding row (Sph, S1P, SK1, *etc*.).

HCV, hepatitis C virus; Sph, sphingosine; S1P, sphingosine-1-phosphate; SK, sphingosine kinase; SPL, sphingosine-1-phosphate lyase; SPP1, sphingosine-1-phosphate phosphatase 1; SPNS2, S1P transporter spinster homolog 2; S1P1, sphingosine 1-phosphate receptor 1; S1P2, sphingosine 1-phosphate receptor 2; S1P3, sphingosine 1-phosphate receptor 3

Supplementary Table 3. Associations between fibrosis stage and mRNA expression or levels of sphingolipid metabolites and the related enzymes in liver tissues in patients without any viruses (n=34).

Variable	Median/Number (1st-3rd Quartile)		*P* values	
	F0-2	F3-4	*P* value	Adjusted *P* value*
Sph (ng/mg)	2.20 (1.37-4.94)	3.91 (0.949-8.55)	0.77	0.51
S1P (ng/mg)	0.249 (0.118-0.419)	0.320 (0.184-0.476)	0.24	0.10
dhS1P (ng/mg)	0.0190 (0.0100-0.500)	0.0410 (0.00500-0.373)	0.79	0.90
SK1 ($\times 1/10^5$)	1.62 (1.22-3.38)	3.25 (2.01-5.60)	0.06	0.06
SK2 ($\times 1/10^5$)	0.931 (0.791-1.76)	1.16 (0.956-1.77)	0.65	0.83
SPL ($\times 1/10^4$)	3.77 (2.59-4.64)	3.52 (2.26-4.49)	0.82	0.67
SPP1 ($\times 1/10^4$)	6.57 (4.89-8.27)	5.08 (4.11-7.20)	0.31	0.20
SPNS2 ($\times 1/10^5$)	1.87 (1.63-2.53)	2.42 (2.17-3.16)	0.03	0.13

S1P1 (×1/10^4)	4.57 (2.59-6.93)	4.15 (2.92-7.35)	0.99	0.90
S1P2 (×1/10^5)	1.84 (1.19-3.66)	2.27 (1.80-3.03)	0.18	0.56
S1P3 (×1/10^5)	8.70 (5.89-10.8)	11.3 (6.12-25.3)	0.11	0.03

*Adjusted for sex and patients age (independent variables). The dependent variables of each *P* value are the items in the leftmost fields of the corresponding row (Sph, S1P, SK1, *etc*.).

Sph, sphingosine; S1P, sphingosine-1-phosphate; SK, sphingosine kinase; SPL, sphingosine-1-phosphate lyase; SPP1, sphingosine-1-phosphate phosphatase 1; SPNS2, S1P transporter spinster homolog 2; S1P1, sphingosine 1-phosphate receptor 1; S1P2, sphingosine 1-phosphate receptor 2; S1P3, sphingosine 1-phosphate receptor 3

Supplementary Table 4. Patient characteristics of patients with metastatic liver tumors

Parameter	n = 9
Female/Male	0/9
Age (years)	64 (54–73)
Platelet count (×10^4 /μL)	23.2 (17.0–28.3)
Albumin (g/dL)	4.1 (3.8–4.4)
AST (U/L)	19 (18–26)
ALT (U/L)	22 (15–24)
GGT (U/L)	46 (25–56)
ALP (U/L)	247 (174-277)
Total bilirubin (mg/dL)	0.7 (0.6–0.9)
PT (%)	100 (100-100)

Continuous variables were represented as the median with 1st and 3rd percentiles and categorical variables were as number and frequencies (%).

AST: aspartate aminotransferase, ALT: alanine aminotransferase, GGT: γ-glutamyl transpeptidase, ALP: alkaline phospatase, PT: prothrombin time

Supplementary Table 5. Associations between the presence of fibrosis and mRNA expression or levels of sphingolipid metabolites and the related enzymes in liver tissues (n=56).

Variable	Median/Number (1st-3rd Quartile)		*P* values	
	Normal liver	F3-4	*P* value	Adjusted *P* value*
Sph (ng/mg)	2.18 (1.16-5.02)	2.87 (1.08-6.90)	0.79	0.51
S1P (ng/mg)	0.290 (0.137-0.391)	0.299 (0.174-0.478)	0.31	0.11
dhS1P (ng/mg)	0.0190 (0.00675-0.0678)	0.0415 (0.00600-0.327)	0.49	0.92
SK1 ($\times 1/10^5$)	1.70 (0.913-7.04)	3.67 (2.02-6.63)	0.46	0.68
SK2 ($\times 1/10^5$)	1.20 (0.900-1.62)	1.18 (0.871-1.88)	0.88	0.86
SPL ($\times 1/10^4$)	3.81 (0.351-5.30)	3.52 (2.23-4.48)	0.18	0.34
SPP1 ($\times 1/10^4$)	5.01 (3.44-5.98)	5.00 (3.98-6.77)	0.70	0.30
SPNS2 ($\times 1/10^5$)	2.04	2.38	0.20	0.39

	(1.57-2.43)	(1.89-3.68)		
S1P1 (×1/10^4)	3.63	4.47	0.19	0.25
	(2.65-4.41)	(3.02-7.33)		
S1P2 (×1/10^5)	1.24	2.42	0.07	0.24
	(0.996-2.66)	(1.53-3.84)		
S1P3 (×1/10^5)	9.82	9.37	0.39	0.17
	(4.90-10.2)	(5.59-15.8)		

*Adjusted for sex and patients age (independent variables). The dependent variables of each *P* value are the items in the leftmost fields of the corresponding row (Sph, S1P, SK1, *etc*.).

Sph, sphingosine; S1P, sphingosine-1-phosphate; SK, sphingosine kinase; SPL, sphingosine-1-phosphate lyase; SPP1, sphingosine-1-phosphate phosphatase 1; SPNS2, S1P transporter spinster homolog 2; S1P1, sphingosine 1-phosphate receptor 1; S1P2, sphingosine 1-phosphate receptor 2; S1P3, sphingosine 1-phosphate receptor 3

FIGURE LEGENDS

Supplementary Figure 1. Protein and mRNA expression levels of SK1 (A), SPNS2 (B) and S1P2 (C). Low and high levels of each proteins were matched with its mRNA expression levels.

Supplementary Figure 2. Relationship between histological fibrosis stages and mRNA expressions of SK1, SPNS2, and S1P2 in patients with hepatitis B (A), with hepatitis C (B), or without hepatitis visuses (C). SK1, SPNS2, and S1P2 mRNA expressions were higher in patients with fibrosis stages 3-4 compared to 0-2 in all etiologies.

A

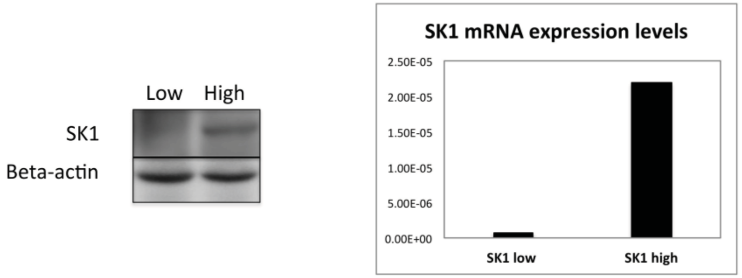
Low
High
SK1
Beta-actin
SK1 mRNA expression levels
2.50E-05
2.00E-05
1.50E-05
1.00E-05
5.00E-06
0.00E+00
SK1 low
SK1 high

B

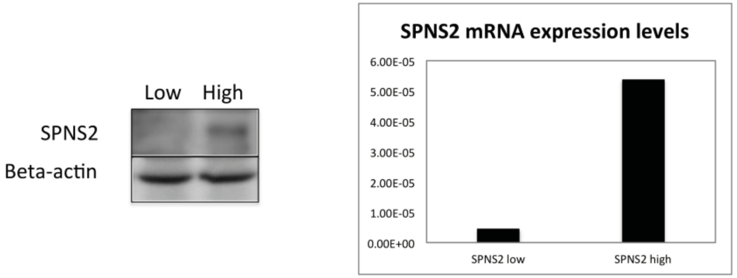
Low
High
SPNS2
Beta-actin
SPNS2 mRNA expression levels
6.00E-05
5.00E-05
4.00E-05
3.00E-05
2.00E-05
1.00E-05
0.00E+00
SPNS2 low
SPNS2 high

C

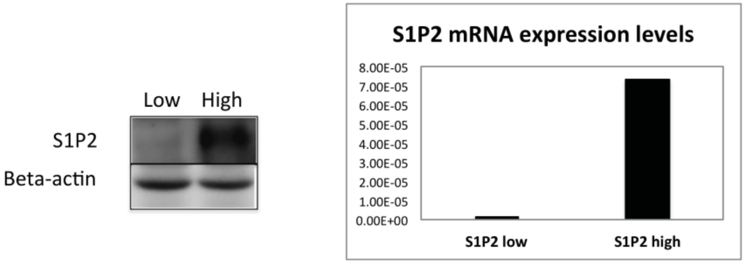
Low
High
S1P2
Beta-actin
S1P2 mRNA expression levels
8.00E-05
7.00E-05
6.00E-05
5.00E-05
4.00E-05
3.00E-05
2.00E-05
1.00E-05
0.00E+00
S1P2 low
S1P2 high

A

B

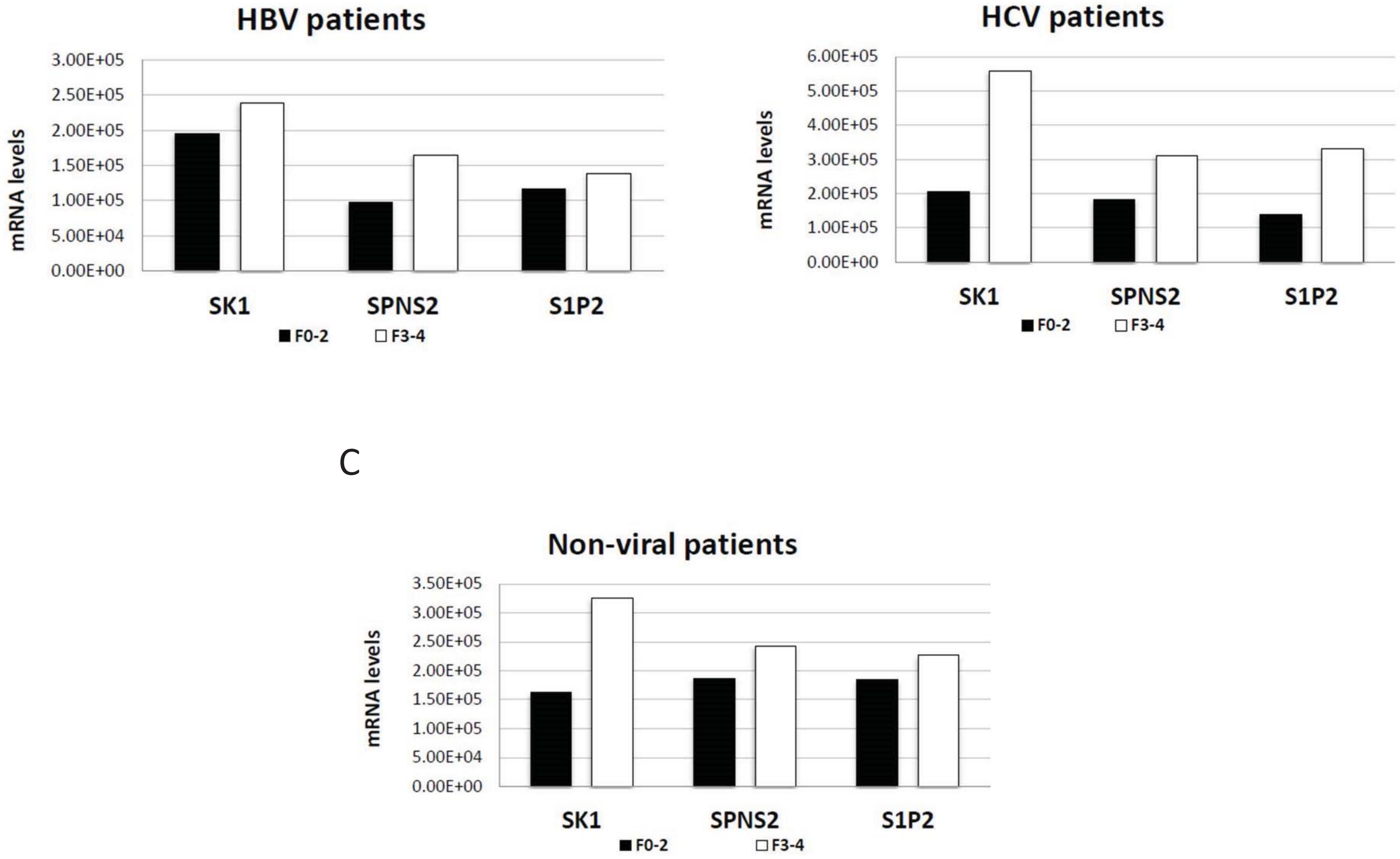
HBV patients
3.00E+05
2.50E+05
2.00E+05
1.50E+05
1.00E+05
5.00E+04
0.00E+00
mRNA levels
SK1
SPNS2
S1P2
F0-2
F3-4
HCV patients
6.00E+05
5.00E+05
4.00E+05
3.00E+05
2.00E+05
1.00E+05
0.00E+00
mRNA levels
SK1
SPNS2
S1P2
F0-2
F3-4
C
Non-viral patients
3.50E+05
3.00E+05
2.50E+05
2.00E+05
1.50E+05
1.00E+05
5.00E+04
0.00E+00
mRNA levels
SK1
SPNS2
S1P2
F0-2
F3-4
